# Vaginal hysterectomy and transvaginal natural orifice transluminal endoscopic surgery for uterosacral ligament suspension for pelvic organ prolapse: 53 cases of single-surgeon experience

**DOI:** 10.1590/1806-9282.20240759

**Published:** 2024-11-11

**Authors:** Murat Ekin, Sukru Yildiz, Aysun Fendal Tunca, Yagmur Yucebas Yildiz, Berk Gursoy, Kardelen Basli Kasim, Keziban Dogan, Cihan Kaya

**Affiliations:** 1 University of Health Sciences Turkey, Bakirkoy Dr. Sadi Konuk Training and Research Hospital, Department of Obstetrics and Gynecology – Istanbul, Turkey.; 2 Istanbul Aydın University, Department of Obstetrics and Gynecology – Istanbul, Turkey.

**Keywords:** Gynecologic surgery, Minimally invasive surgical procedures, Natural orifice transluminal endoscopic surgery, Pelvic organ prolapse, Vaginal hysterectomy

## Abstract

**OBJECTIVE:**

The objective of this study was to describe the single-surgeon experience on transvaginal natural orifice transluminal endoscopic surgery for uterosacral ligament suspension in patients with severe prolapse who had concomitant vaginal hysterectomy.

**METHODS:**

A total of 53 patients with severe uterine prolapse who underwent vaginal hysterectomy and transvaginal natural orifice transluminal endoscopic surgery for uterosacral ligament suspension between January 2021 and March 2023 were included in the study. Operation time, intraoperative and postoperative complications, de novo urinary continence, and duration of hospitalization were obtained from the patient records. Initial postoperative follow-up visits were scheduled for the first week of the month. Patients were followed up yearly, and they had the opportunity to reach the surgical team at any time. Symptomatic prolapse beyond the hymen is defined as recurrence.

**RESULTS:**

Patients had a mean age of 61.7 years ±7.7 SD. All patients received bilateral opportunistic salpingectomy and salpingo-oophorectomy. The total operation time was 162±31 min, with transvaginal natural orifice transluminal endoscopic surgery taking 32.3±5.37 min. There were no intraoperative complications. 12 patients had recurrence; 8 anterior, 3 apical, and 1 posterior prolapse. The mean recurrence time was 11.5 months (range 5–23 months). The reoperation rate was 13.2% (n:7). Three of the patients had obliterative vaginal surgery, three of the patients had anterior, and one patient had posterior repair. Overall failure of apical surgical procedure was 5.6%. Two patients had de novo incontinence postoperatively.

**CONCLUSIONS:**

Transvaginal natural orifice transluminal endoscopic surgery uterosacral ligament suspension is a feasible technique to treat severe pelvic organ prolapse with promising results for short-term efficacy and safety in patients who had concomitant vaginal hysterectomy. Longer follow-up periods are needed to evaluate the long-term efficacy profile of transvaginal natural orifice transluminal endoscopic surgery for uterosacral ligament suspension.

## INTRODUCTION

Pelvic organ prolapse (POP) is a common condition in elderly women that has an impact on quality of life. In total, 11–19% of women will undergo surgery for POP or incontinence by age 80–85 years, and 30% of these women will be estimated to require an additional POP or incontinence surgery^1-3^. After the Food Drug Administration's (FDA) warning statement related to mesh complications, transvaginal mesh repair products were withdrawn from the market in April 2019, and native tissue repair techniques increasingly regained the attention of pelvic reconstructive surgeons^4^.

Transvaginal uterosacral ligament suspension (USLS) is an option as a surgical procedure carried out without mesh, but this procedure has a high risk of ureteral obstruction^5-7^. Transvaginal natural orifice transluminal endoscopic surgery (vNOTES) is described as a procedure with less pain and better cosmetic results compared to abdominal laparoscopic ­surgery^8-10^. Recently, vNOTES for USLS became a treatment option in patients with subtotal or total uterine prolapse who had concomitant vaginal hysterectomy. This procedure also has the advantage of visualization of the ureters, rectum, and adnexa^11,12^. However, at present, there is still a lack of robust evidence on vNOTES pelvic prolapse surgery.

Therefore, we aimed to report the short-term outcomes and complications of vNOTES USLS of a single-surgeon experience.

## METHODS

This retrospective study was approved by both our Institutional Review Board and Local Ethics Committee with approval ­number: 2023-404. We have collected data on cases of vNOTES-­assisted USLS at the Obstetrics and Gynecology Clinic of Bakirkoy Dr. Sadi Konuk Hospital of University of Health Sciences Turkey between January 2021 and March 2023. Detailed informed consent was obtained from all patients. Inclusion criteria: age 40–80 years, no prior pelvic organ prolapse surgery, stage 2–3 apical prolapse, desire to maintain coital function, and refusal of transabdominal repair or transvaginal mesh implantation. Exclusion criteria: severe adhesions from past pelvic surgery, deep infiltrating endometriosis, inability to tolerate the Trendelenburg position or pneumoperitoneum, and suspected gynecological malignancy.

All patients underwent standard vaginal hysterectomy. The operations were carried out by a single surgeon (M.E.) ­experienced with pelvic organ prolapse and endoscopic surgery. The cervix was grasped with a tenaculum and pulled downward, then a circumferential cervical incision was performed. After the dissection of vesicovaginal and rectovaginal fascia with blunt and sharp dissection, posterior and anterior colpotomies were performed, respectively. Bilateral sacrouterine ligaments, cardinal ligaments, uterine arteries, and utero-ovarian ligaments were grasped with Heaney clamps, sealed, cut, and tied with absorbable polyglactin sutures, respectively.

**Figure 1:**
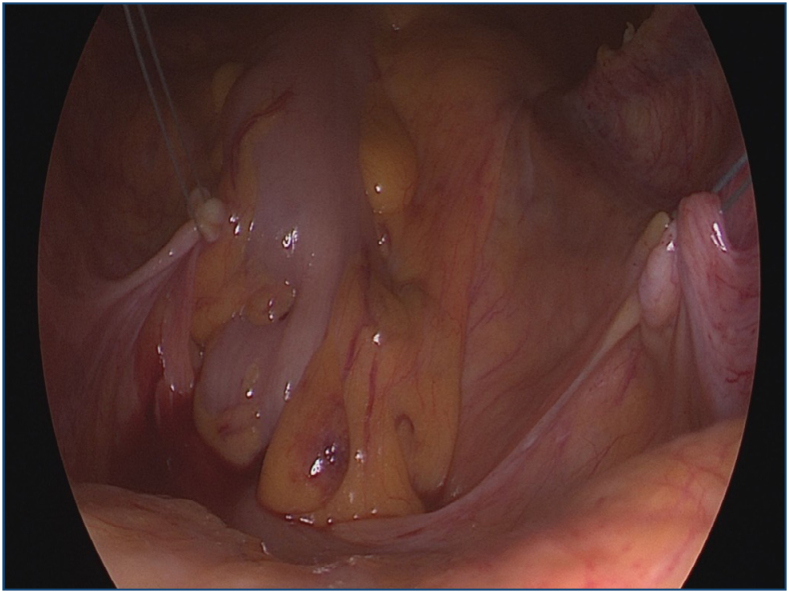
Bilateral sacrouterine ligament sutures on tension.

After uterus removal, a Gel POINT V Path transvaginal access platform including an Alexis wound protector/retractor and Gel Seal cap (Applied Medical, Rancho Santa Margarita, CA, USA) was used for vNOTES uterosacral ligament suspension. All the procedures were performed with the patient in the lithotomy position in a 30° Trendelenburg position under ­general anesthesia. After pneumoperitoneum with 12–18 mmHg CO_2_ insufflation was achieved, a 10-mm rigid 0°–30° telescope was inserted for optical imaging (Visualization System; Karl Storz, Tuttlingen, Germany). Laparoscopic devices such as atraumatic graspers, a needle holder, and an advanced bipolar coagulation-vessel sealing system were used during the procedure. Laparoscopic visualization of the pelvis rectosigmoid, adnexal structures and bilateral ureters was performed. If the ureter was found to be very close to the uterosacral ligament where the sutures are planned to be placed, a peritoneal release incision was performed to avoid kinking of the ureter. Then two 2–0 non-absorbable polyethylene terephthalate double needle sutures (Ethibond) were inserted into the abdomen through the 5-mm trocar ipsilateral to uterosacral ligament. The bilateral uterosacral ligaments were grasped from the caudal segments, pushed gently upward, and the sutures placed around the high-intermediate portion of the uterosacral ligament at the level of the ischial spine bilaterally for a total of 4 stitches (Figure 1), and the free tips of the sutures were pulled down through the ipsilateral previously inserted 5-mm port at Gel Seal cap. The procedure is usually finalized after bilateral removal of the adnexa. The Gel Seal cap and Alexis wound protector/retractor were removed, the posterior and anterior ­vaginal cuff was grasped with Allis clamps, and the sutures were transected bilaterally from the pubocervical fascia posteriorly and anteriorly at the vaginal cuff. The vaginal cuff was closed continuously with absorbable 1–0 polyglactin sutures, and the suspension sutures were tied while the vaginal cuff was pushed upwards as high as possible, suspending the vaginal cuff. After each procedure, cystoscopy was performed to visualize the integrity of the bladder and the jet urine flow from the ureteral orifices to exclude ureteral kinking related to the procedure. Concomitant opportunistic adnexal removal (salpingectomy, salpingo-­oophorectomy) was performed bilaterally. Anterior repair, posterior repair, and perineoplasty were also added to surgery if needed. The total operation time consisted of the vaginal hysterectomy time, USLS time, and the time required for the other ­concomitant procedures. Operation time for vNOTES was defined as the time between insertion of Alexis protector into the peritoneal cavity and take-out time, ­including USLS and the salpingo-oophorectomy or salpingectomy.

The information about patient demographics (age, menopausal status, body mass index, parity), perioperative data included the operative time, blood loss, intraoperative complications (transfusion or injury), postoperative complications (blood transfusion, infection, fever, urinary retention, persistent pain, hematoma formation, de novo stress urinary incontinence), and length of postoperative hospital stay were determined from the patient's electronic files. Postoperative follow-up visits were scheduled for the first week and the first month after surgery. Then patients had a follow-up time yearly, and they had the opportunity to reach the surgical team if they have a complaint of prolapse at any time. The postoperative follow-up of patients and the measurement of POP-Q points were recorded by an experienced gynecologist. Any prolapse POP-Q points zero were recorded as recurrence.

The data were described with mean, standard deviation, median, frequency, minimum, and maximum. Statistical ­evaluation was performed using the SPSS (version 20.0; SPSS Inc., Chicago, IL, USA) software.

## RESULTS

During the study period, 53 patients underwent vaginal hysterectomy and vNOTES for USLS. None of the patients had a prior pelvic organ prolapse surgery. The patient characteristics are shown in Table 1. A total of 26 patients (49%) had stage III prolapse, and the other 27 patients (51%) had stage IV prolapse. 49 of the patients were in the postmenopausal period. The mean age of the patients was 61.7 years±7.7 SD (median 62 years). The operation was successful in all of the cases. Cystoscopy was performed to visualize the integrity of the bladder and the jet urine flow from the ureteral orifices in all of the cases. Bilateral jet urinary flow was observed in all the procedures. No bladder injury was observed. Concomitant opportunistic salpingectomy n=4 (premenopausal patients) and salpingo-oophorectomy n=49 (postmenopausal patients) were performed bilaterally. A total of 22 patients had anterior, and 10 patients had posterior repair. Perineoplasty (n=53) was also performed in all of the cases. The mean total operation time was 162±31 min, and the VNOTES operation time was 32.3±5.37 min.

**Table 1:** Demographic and clinical characteristics of the patients.

Patient's characteristics	Values	n %
Number of patients	53	
Parity (n)	3 (1–13)	
Menopausal status	Premenopausal	4 (5.6%)
	Postmenopausal	49 (94.4%)
Age (years)	61.7±7.7	
BMI (kg/m^2^)	33.2±2.8	
Baseline POP-Q stage	Stage III	26 (49%)
	Stage IV	27 (51%)
Preoperative urinary incontinence	Stress UI	2
	Urge UI	1
	Mixed UI	1

Results are given as mean±standard deviation. BMI: body mass index; POP-Q: pelvic organ prolapse qualification; UI: urinary incontinence.

The mean follow-up period was 10.4 months (4–32). The mean total operation time was 162±31 min, and the mean vNOTES time was 32±6 min. The mean hospital stay was 2.8 days (median 3 days). There were no intraoperative complications. One patient had pulmonary dysfunction. A total of 12 patients had recurrence; 8 anterior (5 stage II and 3 stage III), 3 apical (stage III), and 1 posterior prolapse (stage II). The mean recurrence time was 11.5 months (range 5–23 months). The reoperation rate was 13.2% (n:7). Three of the patients had obliterative vaginal surgery, three of the patients had anterior, and one patient had posterior repair. Overall failure of apical surgical procedure was 5.6% (n:3). In patients with anterior recurrence, the cuff point was between −4 and 0 (median −3). Postoperative de novo urinary incontinence was recorded in two of the patients. Surgical and postoperative evaluations are shown in Table 2.

**Table 2:** Surgical and postoperative evaluation of patients.

Patient's characteristics	Values (n)	
Intraoperative hysterectomy	53	
Concurrent surgery		
Anti-incontinence surgery	0	
Salpingectomy (bilateral)	4	
Salpingo-oophorectomy (bilateral)	49	
Anterior repair	22	
Posterior repair	10	
Perineal body repair	53	
Total operative time (min)	162±31	
vNOTES operative time (min)	32±6	
Preoperative hemoglobin (g/dL)	12.6±1.5	
Postoperative hemoglobin (g/dL)	11.2±1.3	
Postoperative hospital stays (days)	1–6	
Postoperative point C (cm)	-6.47±0.8	
Patients-related complications	1	
Intraoperative complications injury		
Transfusion	0	
Injury to bladder ureters rectum	0	
De novo SUI	2	
Follow-up duration (months)	10.4 (4–32)	
Recurrence at follow-up	Recurrence	Reoperation
Anterior compartment	8 (15.9%)	3 (5.6%)
Posterior compartment	1 (1.8%)	1 (1.85)
Apical compartment	3 (5.6%)	3 (5.6%)
Total	12 (22.6%)	7 (13.2%)

Results are given as mean±standard deviation. vNOTES: transvaginal natural orifice transluminal endoscopic surgery; SUI: stress urinary incontinence.

## DISCUSSION

The utilization of the vNOTES technique in pelvic organ prolapse practice is emerging. There are few studies about vNOTES sacrocolpopexy, which is a relatively complex procedure compared to vNOTES USLS in the literature^9,10^. The vNOTES USLS procedure has been introduced with an improved visualization of important pelvic structures that allows avoidance of surgical complications, including ureteral injury and obstruction^11^.

There are few studies regarding the vNOTES USLS procedure, defining the surgical technique and comparing the intraoperative and early postoperative data with standard USLS. In a pilot retrospective study, Lu et al. reported the short-term outcome of vNOTES USLS in 35 cases^13^. They reported significant improvement in anatomical aspects and quality of life scores with no recurrence at the follow-up period of 1–13 months. The main difference of this study from the current study was the preservation of the uterus in 34.3% of the patients. The follow-up period was also shorter at 3.9±3.8 months, and no recurrence was reported. Postoperative hospital stay was 3.7±1.1 days as with the same with our study.

In a retrospective study, Aharoni et al. compared the surgical and short-term postoperative outcomes of vNOTES USLS versus conventional vaginal USLS in 135 patients^14^. In this study, 65 of the patients had vNOTES surgery. They have concluded that vNOTES hysterectomy with USLS is associated with reduced incidence of intraoperative complications, shorter surgical and anesthesia time, and a slightly longer hospital stay compared to the traditional vaginal surgical approach^14^. The critical point of this study was the higher rate of concomitant adnexal surgery as was performed in all vNOTES procedures and in only 27% of conventional vaginal approaches. Intraoperative complications, including ureteral obstruction, were significantly higher, with a rate of 8% in the conventional USLS group. There was no ureteral obstruction in the vNOTES group. The limitation of this study is the lack of follow-up and short-term results of the vNOTES surgical procedures, including the recurrence rate. Although we do not have a control group, we have similar findings in adnexal surgery rate, hospital stay, and complication rates compared with the study by Aharoni et al^14^. In our study, the long operation time is varied based on additional procedures such as cystoscopy, opportunistic salpingectomy or salpingo-oophorectomy, anterior or posterior repair, and perineoplasty.

vNOTES USLS offers native tissue prolapse repair but may not be effective for advanced cases such as high prolapsed bulky organ volume with a 5.6% apical failure rate. Management of anterior prolapse remains challenging due to high recurrence rates, with eight cases experiencing recurrence possibly due to insufficient preoperative diagnosis of the defect (paravaginal, central, superior, or combined).

In a study, support of the vaginal apex eliminated anterior vaginal wall laxity in 63% of patients with severe apical prolapse, and other studies also reported that more than 70% of anterior wall prolapse could be related to loss of uterine or apical vaginal prolapse^15-17^. Descensus and relaxation of the cuff to the hymenal ring may lead to the recurrence of paravaginal cystocele or previously untreated central cystocele. In our patients with anterior recurrence, the cuff point was between −4 and 0 (median −3) lower than the early postoperative cuff POP-Q point. In our study, five of the anterior recurrent prolapse patients did not want to have a surgical procedure. Three of them have central cystocele and have an anterior repair procedure.

The type of surgery may also affect the postoperative recovery and the success rates of the surgery. In a recent study, Tormena et al. evaluated the inflammatory process in multiport and single port hysterectomy cases, and the authors concluded that there was no significant difference regarding tissue inflammatory response and postoperative pain^18^. However, in vaginal surgery, there is no abdominal incision, less Trendelenburg position requirement, and less duration of surgery compared to abdominal procedures^19^. Further studies should compare abdominal and vaginal approaches, considering not only visual pain scores but also inflammatory markers.

The limitation of our study could be the low number of patients and the retrospective design of the study. However, having the data of one experienced surgeon from a single center and presenting the follow-up results of the patients could be considered the strengths of our study.

## CONCLUSION

As an alternative tissue repair for pelvic organ prolapse, our short-term results of vNOTES USLS are promising, but long-term results from additional studies with larger study populations are still needed. The proper diagnosis of anterior wall prolapses and concomitant anterior wall support is crucial to prevent any postoperative POP complaints.

## Data Availability

The datasets used and/or analyzed during the current study are available from the corresponding author upon reasonable request.
